# Measuring Compounds in Exhaled Air to Detect Alzheimer's Disease and Parkinson’s Disease

**DOI:** 10.1371/journal.pone.0132227

**Published:** 2015-07-13

**Authors:** Jan-Philipp Bach, Maike Gold, David Mengel, Akira Hattesohl, Dirk Lubbe, Severin Schmid, Björn Tackenberg, Jürgen Rieke, Sasidhar Maddula, Jörg Ingo Baumbach, Christoph Nell, Tobias Boeselt, Joan Michelis, Judith Alferink, Michael Heneka, Wolfgang Oertel, Frank Jessen, Sabina Janciauskiene, Claus Vogelmeier, Richard Dodel, Andreas Rembert Koczulla

**Affiliations:** 1 Department of Neurology, RWTH Aachen, 52074 Aachen, Germany; 2 Department of Neurology, Philipps-University Marburg, 35043 Marburg, Germany; 3 Department of Internal Medicine, Division of Pulmonary Diseases, Philipps-University Marburg, 35043 Marburg, Germany; 4 Department of Psychology, Division of Methodology and Statistics of the University of Giessen, 35394 Giessen, Germany; 5 Faculty of Applied Chemistry, Reutlingen University, 72762 Reutlingen, Germany; 6 Clinical Neuroscience Unit, Department of Neurology, University of Bonn, 53105 Bonn, Germany; 7 Department of Psychiatry, University of Bonn, 53105 Bonn, Germany; 8 Department of Psychiatry, University of Münster, 48149 Münster, Germany; 9 German Centre for Neurodegenerative Disease (DZNE), 53105 Bonn, Germany; 10 Department of Internal Medicine, University of Hannover, 30625 Hannover, Germany; Inserm U837, FRANCE

## Abstract

**Background:**

Alzheimer’s disease (AD) is diagnosed based upon medical history, neuropsychiatric examination, cerebrospinal fluid analysis, extensive laboratory analyses and cerebral imaging. Diagnosis is time consuming and labour intensive. Parkinson’s disease (PD) is mainly diagnosed on clinical grounds.

**Objective:**

The primary aim of this study was to differentiate patients suffering from AD, PD and healthy controls by investigating exhaled air with the electronic nose technique. After demonstrating a difference between the three groups the secondary aim was the identification of specific substances responsible for the difference(s) using ion mobility spectroscopy. Thirdly we analysed whether amyloid beta (Aβ) in exhaled breath was causative for the observed differences between patients suffering from AD and healthy controls.

**Methods:**

We employed novel pulmonary diagnostic tools (electronic nose device/ion-mobility spectrometry) for the identification of patients with neurodegenerative diseases. Specifically, we analysed breath pattern differences in exhaled air of patients with AD, those with PD and healthy controls using the electronic nose device (eNose). Using ion mobility spectrometry (IMS), we identified the compounds responsible for the observed differences in breath patterns. We applied ELISA technique to measure Aβ in exhaled breath condensates.

**Results:**

The eNose was able to differentiate between AD, PD and HC correctly. Using IMS, we identified markers that could be used to differentiate healthy controls from patients with AD and PD with an accuracy of 94%. In addition, patients suffering from PD were identified with sensitivity and specificity of 100%. Altogether, 3 AD patients out of 53 participants were misclassified. Although we found Aβ in exhaled breath condensate from both AD and healthy controls, no significant differences between groups were detected.

**Conclusion:**

These data may open a new field in the diagnosis of neurodegenerative disease such as Alzheimer’s disease and Parkinson’s disease. Further research is required to evaluate the significance of these pulmonary findings with respect to the pathophysiology of neurodegenerative disorders.

## Introduction

Alzheimer’s disease (AD) is characterised by the presence of amyloid plaques and neurofibrillary tangles. Plaques mainly comprise extracellular deposits of amyloid-beta (Aβ) [1], including both fibrils and non-fibrillary forms of the peptide [2]. Diagnosing Alzheimer´s disease (AD) based on clinical evidence is difficult. Therefore, surrogate markers have been extensively investigated [3]. Measurements of amyloid beta (Aβ), tau protein and phosphorylated tau protein in cerebrospinal fluid (CSF) have become established fluid biomarkers for making a diagnosis of AD [4, 5]; however, a classification function is required, the method is considerably invasive and the specificity is rather low (sensitivity: 91%; specificity: 64%) [6, 7]. Similarly, magnetic resonance imaging is able to visualise hippocampal and entorhinal atrophy, which is usually progressive throughout the course of the disease [8]. Overall, the diagnosis of AD requires an extensive work up, is time expensive, invasive and usually requires memory clinics. Therefore, identifying an easy to use technique as a screening tool to identify patients with cognitive deficits is useful. Recently, two non-invasive and easily applied technologies have been developed and increasingly used to detect lung disorders and other diseases [9, 10]. Their discriminative ability is derived from specific pattern recognition. Exhaled breath (EB) and exhaled breath condensate (EBC) has been analysed to obtain surrogate information on pulmonary inflammatory processes. EB contains a mixture of volatile organic compounds (VOCs). These compounds can be detected using an electronic nose (eNose), which contains chemical sensors and was originally developed to detect explosives, food contaminations and other compounds. In addition, an eNose enables instant recognition of complex VOC mixtures via composite nanosensor arrays in combination with learning algorithms [10–14]. Each sensor within the instrument reacts differently to a particular VOC mixture, thereby producing a unique pattern. Data analysis follows a heuristic approach, enabling distinctions among “smell-prints” from various sources based on pattern recognition algorithms. The advantages of this technique include its sensitive discrimination performance, its short response time and the reversible behaviour of its sensors [11]. Ion-mobility spectrometry (IMS) [15] has also been employed in this context. This apparatus enables identification of the ions potentially representative of the differences between disorders. Interestingly, a recent publication by Tisch and colleagues [16] reported the application of another method, a nanomaterial based sensors to differentiate patients with Parkinson’s disease (PD), those with AD and healthy controls (HC).

The primary aim of the current study was the evaluation of the eNose to differentiate patients with AD, those with PD and controls. The secondary aim was to identify the compounds responsible for the observed differences between AD, PD and HC using IMS. Furthermore, we analyzed whether the observed differences between patients suffering from AD and HC can be attributed to Aβ in exhaled breath.

## Materials and Methods

### Study participants

We included patients with an established diagnosis of AD and age-matched controls (HC) from two university hospitals in Marburg and Bonn. All of the patients were recruited from the dementia outpatient centres in these two clinics and had previously received a diagnosis of AD. The initial diagnosis of AD was made according to the DSM-IV criteria. However, for the purpose of this study, we also confirmed that the diagnosis remained valid, if the NINDS-ADRDA criteria were applied instead [5]. All of the patients routinely underwent an extensive neuropsychiatric examination [17], cerebral imaging and extensive laboratory screening. In addition, CSF data were considered if available. HCs were also recruited in both centres. Only individuals with no known history of chronic inflammatory disease, tumours or pulmonary disorders were included. From HC a detailed history was taken and cognitive testing was performed using the MMSE. In addition, to test specificity, we included a group of patients suffering from PD. These patients were recruited at the Parkinson Centre of Excellence at the University in Marburg in an outpatient setting. The diagnosis of PD was made based on clinical findings according to the Queens Square Brain Bank Criteria [18]. A movement disorder specialist evaluated all patients. and in most of the patients a FP-CIT SPECT was present to additionally support the diagnosis. However, patients whose diagnosis was uncertain were excluded.

The study was carried out in accordance with German law and international guidelines for clinical studies. The study was approved by the local ethics committee (Marburg Ethics Committee AZ 107/08; 139/11 and Bonn Ethics Committee AZ 311/11), and written informed consent was obtained from each subject before enrolment in the study.

### Electronic nose

For VOC sampling, the Cyranose 320 (C-320) eNose (Smiths Detection Group Ltd., Watford, UK) was used. This instrument is a hand-held device capable of generating so-called smell-prints by analysing mixtures of VOCs as described previously [19]. The participants breathed standardised medicinal air (Aer medicinalis Linde, Linde Gas Therapeutics GmbH, Unterschleißheim, Germany) and then exhaled for ten seconds at a flow rate of 100–200 ml/s into a collection bag [20]. All of the measurements were performed in triplicate.

### Ion mobility spectrometry

We used a BioScout IMS (B&S Analytik, Dortmund, Germany) coupled to a multi-capillary column (MCC/IMS) and a SpiroScout spirometer (Ganhorn Medizin Electronic, Niederlauer, Germany) as the sample inlet unit, as described previously [15]. All of the subjects were asked to exhale through a mouthpiece connected to a Teflon tube. The VOC peaks were characterised using the software Visual Now 2.2 (B&S Analytik, Dortmund, Germany), which is described elsewhere [15, 21]. All of the detected peaks were characterised according to the drift time (corresponding 1/K_0_-value), retention time and concentration (represented by the peak height). A preliminary relationship between the peak position and the identity of the analyte was obtained using the database BSIMSDB 1.4 (B&S Analytik, Dortmund, Germany).

### Lung tissue protein lysates

For animal studies, we used four heterozygous adult female transgenic CRND8 mice expressing mutant APP Swedish (K670N,M671L) and mutant APP Indiana (V707F) under the control of a hamster prion promoter on a hybrid Hybrid C3H/He-C57BL/6 background. Four gender-matched non transgenic wild-type mice served as control population. Mice were housed on a 12 h light-dark schedule (lights on 07:00–19:00). They had free access to tap water, were fed ad libitum and kept under standard conditions. Animal procedures were approved by the office of the federal state authority of Hessen and the Institutional Animal Care and Use Committee of the University of Marburg (AZ V54-19c20-15(1) MR20/15—Nr. 6/2008) as well as by the institutional animal welfare officer (AZ AK-4-2014-DODEL). All experiments were carried out in accordance with EU Directive 1020/63/EU for the protection of animals used for scientific purposes. Lung tissue samples were washed extensively in ice-cold phosphate-buffered saline (PBS), minced using a scalpel and dissolved for thirty minutes in radio immunoprecipitation assay (RIPA) buffer containing a protease-inhibiting cocktail (Roche, Germany). The protein concentration of the lysates was determined using a bicinchoninic acid (BCA) assay kit (Pierce, Rockford, IL).

### Exhaled breath condensate

EBC samples were collected during ten minutes of tidal breathing through a single-use disposable RTube device (Respiratory Research Inc., Austin, TX, USA). Immediately after collection, the EBC was transferred into low-bind polyethylene tubes (Eppendorf AG, Hamburg, Germany) and stored at -80°C.

### Western blotting

To detect the AβPP cleavage products C83 andC99 in mice lung tissue, samples were boiled in SDS loading buffer, separated on a NuPage Novex 4–12% Bis-Tris gel (Invitrogen, Camarillo, CA) and then blotted onto a nitrocellulose membrane. Following incubation with a C-terminal AβPP antibody (Sigma Aldrich, Taufkirchen, Germany) and secondary antibody (Vector labs, Burlingame, CA, USA), the membranes were exposed to an autoradiographic film.

### MSD ELISA Aβ_40_ and Aβ_42_

EBC samples from patients with AD and HC, as well as mouse lung tissue samples were analysed for the presence of Aβ_40_ and Aβ_42_ using the MSD 96-well MULTI-SPOT Human (6E10) Aβ Triplex Assay (Meso Scale Discovery, Gaithersburg, MD) according to the manufacturer’s instructions. The assay was conducted in triplicate, and the Aβ concentrations were calculated with respect to a standard curve.

### Data analysis

Linear discriminant analysis (LDA) [22] was used to distinguish among the groups. The first experiment involved distinguishing patients with AD from controls. The output of the 32 sensors of the Cyranose C-320 was factorised using principal component analysis (PCA). To discriminate between groups, we used a combination of the raw values of the C-320 and PCA scores. To identify the variables with the highest predictive value, predictors identified in the LDA models were selected stepwise using the Akaike information criterion. To confirm the validity and stability of the LDA models, we applied two distinct cross-validation strategies. First, a leave-one-out cross-validation (LOOCV) was performed using the data acquired at Marburg and Bonn, separately. For this analysis, both the PCA scores and the LDA models were computed separately within each set of training data and then applied to predict the test data. We then fitted models for the data from one site (Bonn, Marburg) to make predictions for the other site.

In a second approach, patients with PD, AD and HC were distinguished using the eNose. The repeated measurements were evaluated according to median values, as they turned out to be more heterogeneous than expected, and normalised to a range from 0 to 1. As a variance-dependent distance measure for multidimensional data, the Mahalanobis distance (MD)(MD(*x*, *y*) = ((*x*—*y*)^T^ *C* ^-1^(*x*—*y*))^1/2^, with *x* = (*x*_1_, …, *x*_n_), *y* = (*y*_1_, …, *y*_n_) and *C* ^-1^ as the inverse covariance matrix of the *n*-dimensional data) between groups was used. An MD value greater than 1.96 was considered significant, as it corresponds to a p-value of < 0.05, whereas a MD value greater than 2.58 indicated a p-value of < 0.01. A *k*-fold cross-validation (in which *k* equals the product of the group sizes) using one data point of each group as test data in each run was performed to calculate the cross-validation value (CVV) for the experiments.

For IMS analysis, we used the software VisualNow (B&S Analytik GmbH, Dortmund, Germany) to separate between two classes. The best threshold value to separate each analyte separately will be calculated and the classifications obtained are considered. Finally, the sensitivity, specificity, positive and negative predictive value and the accuracy ((true positive + true negative)/(true positive + false positive + true negative + false negative)) are calculated. As result, the separation power of each peak can be obtained. Decision tree learning uses a decision tree as a predictive model which shows maps of observations about an item to find conclusions about the item's target value. Here, all peaks were considered and the shortest way to separate two or more classes is calculated. Class in our examples is equivalent to AD, HC, PD. It is one of the most predictive modelling approaches used in statistics, data mining and machine learning. Tree models where the target variable can take a finite set of values are called classification trees. In these tree structures, leaves represent class labels and branches represent conjunctions of features that lead to those class labels. As for boxplots, also decision trees can be used to separate classes and to calculate sensitivity and specificity values as described above. Only the variables (peaks, analytes) used in the tree are taken into account. Therefore, using decision trees the separation power of all analytes selected is considered in contrast to box-plots looking for the separation power of each peak separately.

ELISA data were compared using a t-est. Mean values and standard error of the mean are presented in all cases.

## Results

We performed eNose assays on 18 patients with AD and 19 HC recruited from the Department of Neurology, University of Marburg. The clinical data for both study populations are described in Table 1.

**Table 1:** Clinical characteristics of patients with Alzheimer’s disease and healthy controls.

		Marburg			Bonn		
	AD^a^	PD^b^	HC^c^	p-value	AD	HC	p-value
Female / male	9 / 9	5/11	11 / 8	n.s^i^	13 / 8	8 / 8	n.s. ^i^
Age [years]	71(10.2)	64 (11.3)	60 (12.8)	n.s^i^	72 (7.4)	67 (8.8)	n.s. ^i^
Age at onset [years]	69 (10.3)	51.8 (18.6)	n/a^d^		70 (7.1)	n/a	
Disease duration [months]	18 (17.6)	7.9(5.2)	n/a		18 (18.4)	n/a	
MMSE^e^ score	22 (3.7)	28 (3.2)	n/a		19 (4.8)	29 (0.9)	
Smoking current / former	1 / 7		3 / 4	0.28	1 / 1	2 / 1	0.28
Drug treatment							
Donepezil	8	0	0		10	0	
Galantamine	1	0	0		6	0	
Rivastigmine	2	0	0		4	0	
Memantine	5	0	0		1	0	
Cerebrospinal fluid taken	5	0	0		17	0	
Tau [pg/ml]	261.00 (105.594)	n/a	n/a		694.30 (390.808)	n/a	
pTau^f^ [pg/ml]	47.00 (13.589)	n/a	n/a		109.38 (44.336)	n/a	
Ratio Aβ_42_ ^g^/Aβ_40_ ^h^	0.06 (0.028)	n/a	n/a		0.07 (0.027)	n/a	

All values are arithmetic means with standard deviations in parentheses, except for sex, smoker status, drug treatment, and cerebrospinal fluid taken.

Abbreviations

^a^ AD Alzheimer’s disease

^b^ PD Parkinson’s disease

^c^ HC healthy control

^d^ n/a not applicable

^e^ MMSE mini-mental state examination.

^f^ pTau hyperphosphorylated tau protein

^g^ Aβ_42_ amyloid-beta 1–42

^h^ Aβ_40_ amyloid-beta 1–40

^i^ n.s. not significant.

Our first aim was to test whether patients with AD and HC could be differentiated according to differences in their breathing patterns using the eNose. We also trained the machine using the “training setting”. The observed pattern was later used to differentiate patients and HC using this technique alone in a different location and on different patients to evaluate the accuracy of the machine.

We found that a combination of the raw values from the 1^st^ sensor and the 22^nd^ principal component showed the best performance in terms of differentiating between patients with AD and HCs. Following statistical analysis, we optimised an LDA model [19] through the stepwise selection of a combination of predictors. Using LOOCV, we found a significant relationship between the predicted and actual group affiliations (*χ* ^2^ = 8.17, degrees of freedom (df) = 1, *p* = 0.004) with a sensitivity of 50.0% (95%-CI: 36.8–63.2) and a specificity of 77.2% (95%-CI: 64.2–87.3) (Table 2).

**Table 2:** LDA^a^ classifications for the leave-one-out cross-validations.

	Marburg				Bonn		
estimated/ true	AD^b^	HC^c^	Sum	estimated/true	AD	HC	Sum
AD	30	13	43	AD	44	15	59
HC	30	44	74	HC	19	33	52
Sum	60	57	117	Sum	63	48	111

Table 2 shows cross tables of the estimated vs. true AD and HC participants from the two sites (Marburg and Bonn). Estimations were drawn from the outcome of a leave-one-out cross-validation of the LDA model.

Abbreviations

^a^ LDA Linear discriminant analysis

^b^ AD Alzheimer’s disease

^c^ HC healthy control

**Fig 1:**
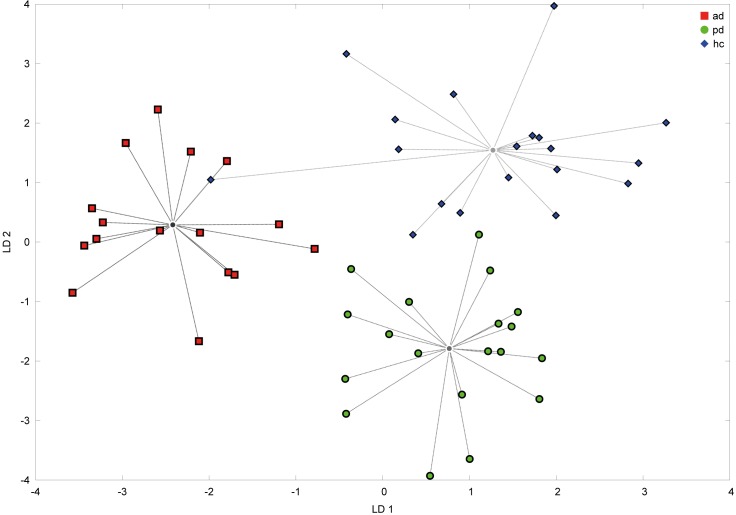
Linear discriminant analysis. In Fig 1, we tested whether differentiating among patients with two neurodegenerative disorders and healthy controls is possible using the eNose. Linear discriminant analysis (LDA) was used to distinguish among groups. Repeated measurements were evaluated using median values and normalised to a range of 0 to 1. LD = linear discriminant, ad = Alzheimer's disease, pd = Parkinson's disease, hc = healthy control.

Considering that it is possible to detect changes in breath patterns using the eNose, we tested whether it would be possible to distinguish patients with AD from those with another neurodegenerative disorder (PD), as well as either from HC. The result is shown in Fig 1. The linear discriminant analysis is able to separate the three groups: patients with AD, patients with PD and age-matched controls, as shown in the canonical plot (Fig 1). The LDA provides excellent performance and reveals significant differences between AD and PD (*p* < 0.0001), AD and HC (*p* < 0.0001), and PD and HC (*p* < 0.0001) with respect to distinctions between groups. The MD between AD and PD is 2.08 (*p* = 0.04); that between AD and HC is 2.10 (*p* = 0.04); and that between PD and HC is 1.95, which is at the borderline of significance (*p* = 0.05). From these results, we concluded that making a specific assessment of neurodegenerative disease is possible.

To validate the results of the training set, we included a second independent cross-sectional group of patients with AD and HC in line with best practice guidelines for biomarker development [23]. For this validation, 21 AD patients and 16 HC were recruited from the Department of Psychiatry, University Hospital Bonn (Table 1). In the second study population, HC could be significantly discriminated from AD patients in an LOOCV (*χ* ^2^ = 14.78, *df* = 1, *p* < 0.001) with a sensitivity of 69.8% (95%-CI: 57.0–80.8) and a specificity of 68.7% (95%-CI: 53.7–81.3) (Table 2).

To confirm that the predictions of the LDA models remained valid beyond their respective sites (Bonn or Marburg), we used the LDA models from the prior analyses from each site and applied it to discriminate participants from the other site. The LDA model fitted with data from Marburg was able to significantly discriminate groups from Bonn (*χ* ^2^ = 5.00, *df* = 1, *p* = 0.025) with a sensitivity of 76.2% (95%-CI: 63.8–86.0) and a specificity of 45.8% (95%-CI: 31.4–60.8). The LDA model fitted with data from Bonn, however, was not able to detect AD patients from Marburg above a chance level (*χ* ^2^ = 0.20, *df* = 1, *p* = 0.658) with a sensitivity of 56.6% (95%-CI: 43.2–69.4) and specificity of 49.1% (95%-CI: 35.6–62.7) (Table 3).

**Table 3:** LDA classifications for the cross-validation between Bonn and Marburg.

	LDA: Bonn to Marburg				LDA: Marburg to Bonn		
estimated/ true	AD	HC	Sum	estimated/ true	AD	HC	Sum
AD	34	29	63	AD	48	26	74
HC	26	28	54	HC	15	22	37
Sum	60	57	117	Sum	63	48	111

Table 3 shows cross tables of the estimated vs. true AD and HC participants. Estimates for the LDA were gathered from one site and applied to classify the participants from the other site (LDA: Bonn to Marburg and LDA: Marburg to Bonn).

**Fig 2:**
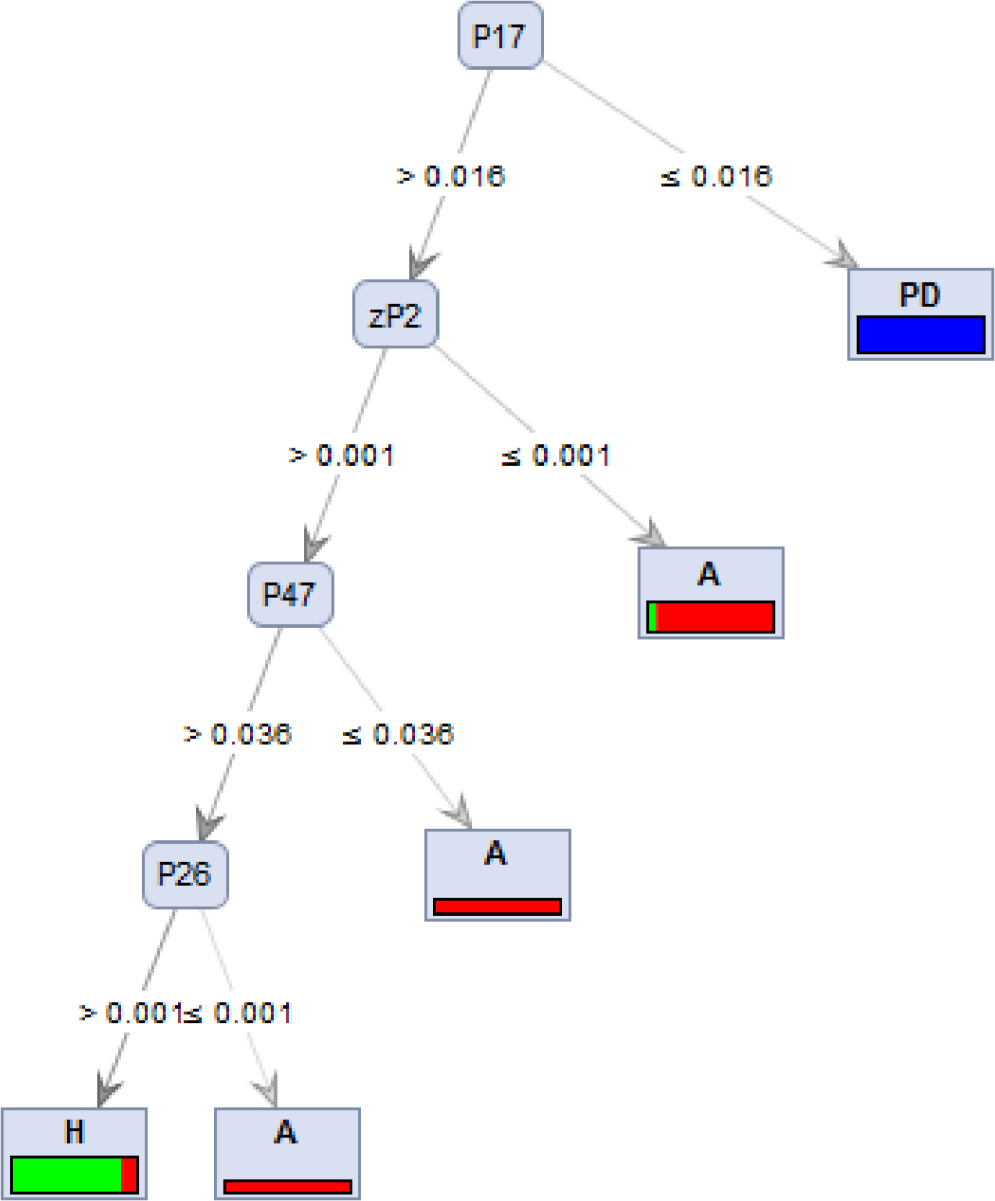
Decision tree of four variables measured using IMS. Exhaled breath from 21 AD, 16 PD patients and 16 HC was analysed using IMS. A decision tree based on four compounds is shown in Fig 2. Samples are grouped according to the means of the peak intensity of each compound, at which point, the maximum number of samples are classified correctly. Relative numbers of classified HC are green, and numbers of classified patients with AD are red. PD is marked in blue. Total numbers of classified samples are given for each compound. P denotes the concentration of a compound. Using a decision tree with four characteristics, the method shows a accuracy of 94% when differentiating patients with AD from HC. Considering PD/AD vs. HC, sensitivity of 95% and specificity of 94%, positive predictive value of 97%, negative predictive value of 88% were calculated.

To further characterise differences between the three groups, we supplemented our diagnostic approach by applying IMS on exhaled air samples. This task was performed on AD patients, PD patients as well as healthy controls. A typical IMS chromatogram for the positive ions of analytes is shown in S1 Fig To discriminate between patients with AD, PD and HC, the optimal thresholds for each analyte were identified. The optimal threshold is the peak intensity that allows the maximum number of samples to be classified correctly. The analysis using IMS detected significant differences in five organic compounds with high specificity and sensitivity. S1 Table shows two of the peaks that were deemed suitable for differentiating between AD patients and HC (S1 Table). Because a range of sensitivity and specificity values was obtained using IMS, we considered an analysis that relied on data mining (calculated using Rapid Miner Version 5.1, Rapid-I, Dortmund, Germany). Decision tree learning is a method commonly used to perform data mining. The aim is to create a model that predicts the value of a target variable based on several input variables. Using a decision tree with four compounds, the method exhibited a considerably high accuracy of 94% (95%-CI 88–100). Only three patients were misclassified of a total of 53 participants (Fig 2). In addition, the decision tree shows a sensitivity and specificity of 100% for PD. Within the three false classifications, one AD patient was declared as HC and two HC were incorrectly identified as AD. All other patients were diagnosed correctly. The first input variable included in the decision tree was 1-butanol (P17) at a threshold of 0.016. All samples with values lower than 0.016 were correctly identified as PD with no false predictions. For all samples with values equal to or greater than 0.016, a second input variable to differentiate between HC and AD was required. The variable zP2 was used for the second differentiation. Samples with values lower than 0.001 were, in six out of seven cases, AD, whereas samples with values equal to or more than 0.001 were compared with respect to the next analyte, 2-methylfuran (P47). In this study, all samples with values lower than 0.036 were correctly identified as AD, whereas all samples with values equal to or greater than 0.036 were compared with respect to the last input variable, an unidentified component (P26). With values below or equal to 0.001, patients with AD were correctly identified. Values greater than 0.001 were identified as healthy controls, with two false allocations. To summarise, we found four substances suitable for differentiating patients with AD from patients with PD as well as from HC. These substances are all organic compounds. It should be mentioned that each analyte *per se* is not suitable for differentiating patients with AD and PD from HC; rather, it is the combination and sequence of analytes that makes prediction feasible. Considering the combination of neurodegenerative disorders (PD/AD) vs. HC, sensitivity of 95% (95%-CI 87–100) and specificity of 94% (95%-CI 82–100) as well as a positive predictive value of 97% and a negative predictive value of 88% were calculated.

In an additional set of experiments, we investigated whether the observed differences between patients with AD and HC with respect to exhaled air can be attributed to spurious detection of Aβ. We do not consider Aβ to be a volatile substance. However, with respect to the pathogenesis of AD, we assumed that the observed difference may be due to changes in peripheral Aβ metabolism. In APP transgenic CRND8 mice Aβ concentration in lung tissue is unchanged compared to wild-type mice (S2A Fig; p > 0.05). In line with this no differences in APP cleavage products C83 and C99 can be observed between both groups of animals (S2B Fig). Using MSD ELISA, we detected Aβ at markedly low concentrations in breath condensate samples of patients with AD and HC, but failed to observe a significant difference between the two groups. (S2C Fig). In summary, we found no evidence for changes in peripheral Aβ metabolism in lungs in an animal model of AD as well as in breath condensates of patients with AD and HC.

## Discussion

We provide evidence that a novel non-invasive diagnostic approach based on the analysis of exhaled air can differentiate patients with Alzheimer’s disease (AD) from those suffering from Parkinson’s disease (PD) and from healthy controls. Using an eNose, we were able to see differences in the breath patterns of AD patients and HC from two independent sites in Germany. These results are in line with those of Tisch et al., who also described differences in breath patterns between patients with AD, those with PD and HC [16]. Using the IMS technique, we obtained a sensitivity of 95% and a specificity of 94% in terms of differentiation between neurodegenerative disorder and HC. It has been shown that evaluating the combination of CSF tau protein, phosphorylated tau protein_181_ and Aβ_42_ achieves a sensitivity of 85% and a specificity of 80% in the identification of AD [24]. Our sensitivity values using IMS (which offers the benefit of being a non-invasive approach) are at least comparable to the aforementioned rates. The inclusion of a decision tree comprising four substances detected using IMS provides even better performance in terms of sensitivity and specificity. Interestingly, although Aβ was detectable at low level in EBC and in lung tissue, we were unable to identify this peptide as a determining factor during the IMS analyses. Furthermore, we could not observe altered levels of Aβ in EBC of patients with AD compared to HC using ELISA technique. The same holds true for the animal model of AD. It is therefore most likely that differences in blood chemistry in response to disease are responsible for the observed changes in exhaled breath. Tisch and colleagues proposed a similar theory [16]. Of note, since the diagnostic accuracy for PD was 100%, it had to be excluded that this peak was due to L-Dopamin or derivatives. P17 is 1-butanol, thus making it less likely. However, in a second step, we analysed the exhaled breath condensate using gas chromatography to check for dopamine concentrations (data not shown). L-dopa and dopamine could not be measured, thus making a confounding effect unlikely.

To the best of our knowledge, little is known about the role of these four volatile substances in neurodegenerative disorders. Interestingly, a prior study reported an association of organic solvents with the development of PD [25]. The authors analysed a group of 99 pairs of twins who were discordant for PD. For trichloroethylene exposure, they found a significantly increased risk of PD. For two other solvents (perchloroethylene and carbon tetrachloride), the association was strong but not significant. The authors also analysed xylol, which was not associated with an increased risk of PD. Interestingly, there is also a rodent model of PD in which the hallmarks of the disease are induced by exposure to trichloroethylene. In a recent publication by Tisch, AD and PD were identified from VOCs in exhaled breath using gas chromatography-mass spectroscopy [16]. They also detected 24 organic components that could be used to distinguish patients from healthy controls. The compounds were present in both patients with AD and HC and increased in AD. Our approach was different; we also describe several VOCs that are reduced in patients with AD compared with HC. In addition, Tisch and colleagues did not report detecting any of our components using IMS. However, it was possible to differentiate patients with AD, PD and HC using a decision tree and four of these substances. Thus, it appears likely that these four substances are specifically involved in disease processes. The roles of these organic substances remain unclear, and it cannot be anticipated whether there is one or more correlations among disease progression, medication, disease development and these substances. It has been hypothesised that oxidative stress leads to cell wall break down and liberation of phospholipids, which in turn can give rise to volatile organic components [16]. Therefore, further research is necessary to investigate the possible roles of these substances in the development of AD and PD.

Despite careful planning, there are some limitations of this study, including the sample size of the groups and the age difference between the two groups. The sample size is a limitation, but the present study was a pilot study in order to see whether it was possible to view differences between these disorders. A larger study is currently planned in order to validate these results in a larger sample size. In this study, correlation of the patterns with alterations in CSF and neuroimaging are considered. In addition, all of the patients in the second study population were taking anti-dementia drugs. It is therefore theoretically possible that all of the organic compounds that we detected are part of the pharmacokinetics of these drugs. However, this possibility is highly unlikely given the structural relationships and known pharmacokinetics of the respective drugs. In addition, the concentration of the measured items was in some cases reduced in the patients and increased in healthy controls, further arguing against this possibility.

In summary, both VOC sampling using the eNose and IMS were successfully able to characterise patients with AD, PD and HC. This diagnostic approach offers a number of advantages in comparison with currently available fluid biomarkers: it can distinguish with high efficacy between AD, PD and HC, it avoids invasive and potentially harmful procedures. Following validation of this approach within a larger patient sample, it may be used as a screening tool with a hand-held device in an ambulatory setting prior to further sophisticated evaluation. In addition, it may be suited for assisting in diagnosis in remote areas, where imaging technics or specialists are scarce.

## Supporting Information

S1 Fig IMS-Chromatogram with peak positions (black and blue crosses). —x-axis: drift time (1/K_0_).—y-axis: retention time through the multi-capillary column.—color: yellow highest intensity, red: middle.
(DOCX)

S2 Fig Aβ concentrations do not differ significantly in exhaled breath condensate between patients with AD and age-matched HC as well as in lung tissue of APP transgenic mice compared to wild-type mice. (a) Mice lung lysates of APP transgenic CRND8 mice (n = 4) as well as wild-type mice (n = 4) were analysed using a MSD Human (6E10) Aβ Triplex Assay; all values are displayed in pg Aβ/mg total protein. (b) For investigation of APP processing, AβPP cleavage products C83 and C99 were analysed by Western blotting. (c) EBC samples were analysed using an MSD Human (6E10) Aβ Triplex Assay. 16 HC and 21 patients with AD were tested. Values are given in pg/ml EBC. Error bars represent the standard deviation of the mean.
(PDF)

S1 Table Example of two organic compounds measured with IMS. Two organic solvents are shown, that can be used to differentiate between AD and HC.
(DOCX)
