# Supplementary figures and images for: Measuring Compounds in Exhaled Air to Detect Alzheimer's Disease and Parkinson’s Disease
Source: PLoS One. 2015 Jul 13;10(7):e0132227. doi: 10.1371/journal.pone.0132227 (PMC4500505; doi:10.1371/journal.pone.0132227)

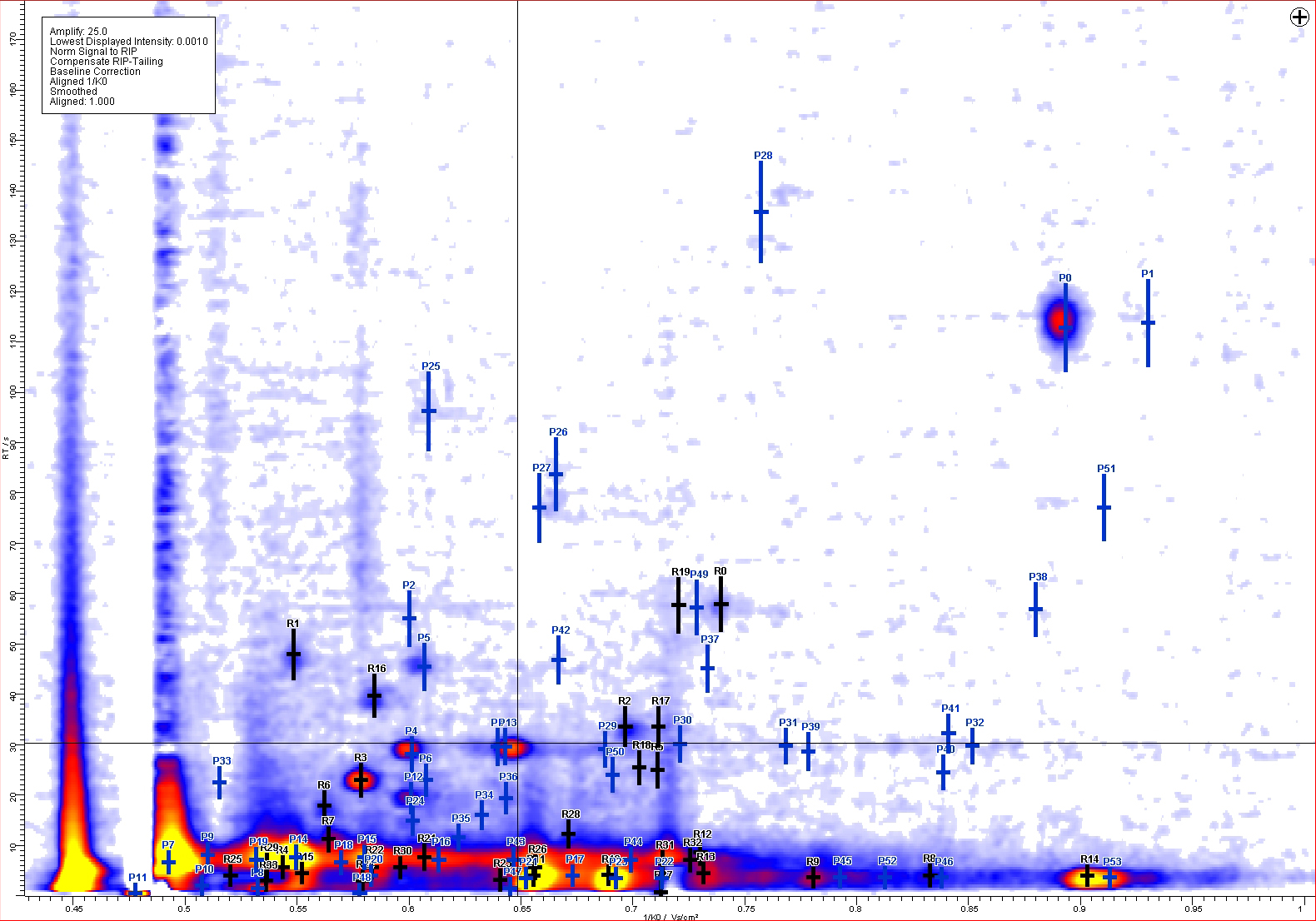

Supplement: S1 Fig — —x-axis: drift time (1/K0).—y-axis: retention time through the multi-capillary column.—color: yellow highest intensity, red: middle. (DOCX) [file pone.0132227.s001.docx]

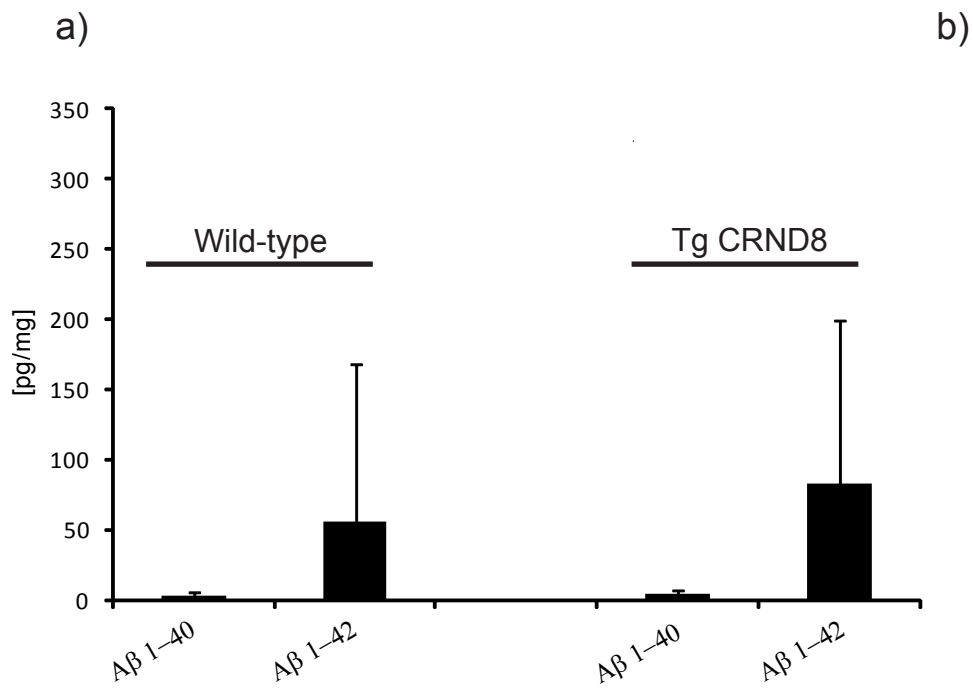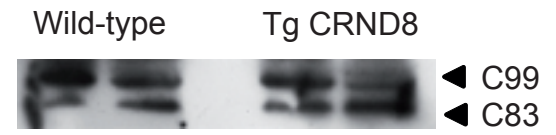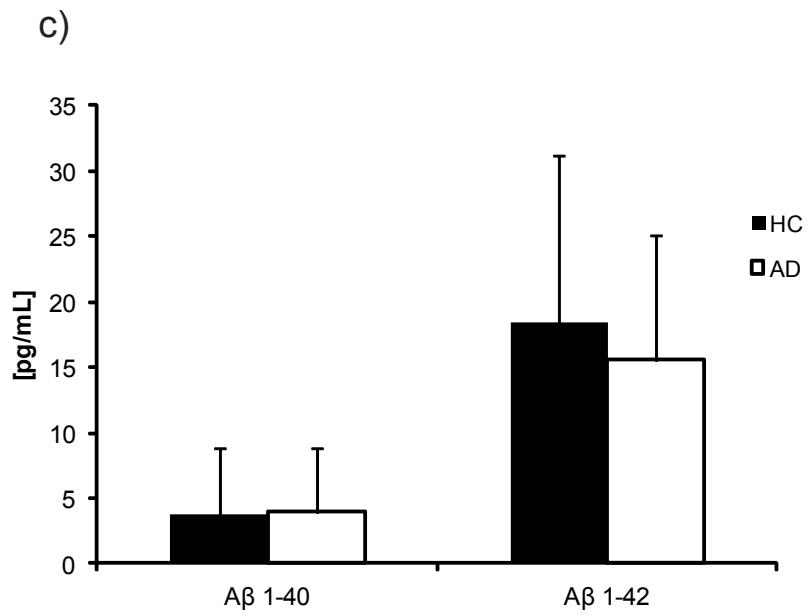

Supplement: S2 Fig — (a) Mice lung lysates of APP transgenic CRND8 mice (n = 4) as well as wild-type mice (n = 4) were analysed using a MSD Human (6E10) Aβ Triplex Assay; all values are displayed in pg Aβ/mg total protein. (b) For investigation of APP processing, AβPP cleavage products C83 and C99 were analysed by Western blotting. (c) EBC samples were analysed using an MSD Human (6E10) Aβ Triplex Assay. 16 HC and 21 patients with AD were tested. Values are given in pg/ml EBC. Error bars represent the standard deviation of the mean. (PDF) [file pone.0132227.s002.pdf]
