# Supplementary material for: Measuring Compounds in Exhaled Air to Detect Alzheimer's Disease and Parkinson’s Disease
Source: PLoS One. 2015 Jul 13;10(7):e0132227. doi: 10.1371/journal.pone.0132227 (PMC4500505; doi:10.1371/journal.pone.0132227)
Supplement: S1 Table — Two organic solvents are shown, that can be used to differentiate between AD and HC. (DOCX) [file pone.0132227.s003.docx]

**Supplemental table 1: Example of two organic compounds measured with IMS**

|  | 2-Ethyltoluene | 3-Octanone |
| --- | --- | --- |
| estimated right | 27 | 27 |
| estimated wrong | 10 | 10 |
| true positive | 8 | 14 |
| false positive | 2 | 8 |
| true negative | 19 | 13 |
| false negative | 8 | 2 |
| Sensitivity | 0.50 | 0.88 |
| Specificity | 0.90 | 0.62 |
| Positive predictive value | 0.80 | 0.64 |
| Negative predictive value | 0.70 | 0.87 |
| significance level | < 0.05 | < 0.05 |
